# Anti‐inflammatory and immunomodulatory effects of *Lactobacillus* spp. as a preservative and therapeutic agent for IBD control

**DOI:** 10.1002/iid3.635

**Published:** 2022-05-19

**Authors:** Shadi Aghamohammad, Amin Sepehr, Seyedeh Tina Miri, Saeideh Najafi, Mohammad R. Pourshafie, Mahdi Rohani

**Affiliations:** ^1^ Department of Bacteriology Pasteur Institute of Iran Tehran Iran; ^2^ Department of Biology, Science and Research Branch Islamic Azad University Tehran Iran

**Keywords:** IBD, *JAK*/STAT, *Lactobacillus* spp., NF‐κB

## Abstract

**Background:**

Probiotics have a beneficial effect on inflammatory responses and immune regulation, via Janus kinase/signal transduction and activator of transcription (JAK/STAT) and NF‐κB signaling pathways. To evaluate the precise effects of *Lactobacillus* spp. as a protective and therapeutic agent, we aimed to investigate the efficacy of *Lactobacillus* spp. in modulating JAK/STAT and nuclear factor kappa B (NF‐κB) inflammatory signaling pathways.

**Methods:**

A quantitative real‐time polymerase chain reaction (qPCR) assay was used to analyze the expression of JAK/STAT and inflammatory genes (TIR‑associated Protein [*TIRAP]*, Interleukin 1 Receptor Associated Kinase[*IRAK4]*, Nuclear factor‐kappa B Essential Modulator [*NEMO]*, and receptor interacting protein [*RIP]*) followed by treatment of the HT‐29 cell line with sonicated pathogens before, after, and simultaneously with *Lactobacillus* spp. A cytokine assay was also used to evaluate interleukin (IL)‐6 and IL‐1β production after treatment with *Lactobacillus* spp.

**Results:**

*Lactobacillus* spp. downregulated JAK and *TIRAP*, *IRAK4*, *NEMO*, and *RIP* genes in the NF‐κB pathway compared to sonicate‐treated cells. The expression of *STAT* genes was different after treatment with probiotics. The production of IL‐6 and IL‐1β decreased after probiotic treatment.

**Conclusions:**

Our *Lactobacillus* spp. cocktail showed anti‐inflammatory effects on HT‐29 cells by modulating JAK/STAT and NF‐κB signaling pathways in all three treatment variants. Therefore, *Lactobacillus* spp. as a dietary supplement can both prevent and reduce inflammation‐related diseases such as inflammatory bowel disease.

## Graphical abstract

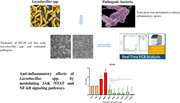

## 1 INTRODUCTION

Probiotics play several important roles in improving health. Modulating the gut microbiota, regulating the immune system, showing anti‐inflammatory effects, and having activities against pathogens by regulating mucus secretion are some of the important functions of probiotics.^1^, ^2^ Lactic acid bacteria, including *Lactobacillus* spp., are the most important and well‐known probiotics. *Lactobacillus plantarum* was first isolated from saliva. Later, it was demonstrated that *L. plantarum* produces hydrogen peroxide and therefore has remarkable antimicrobial activity.^3^, ^4^ *Lactobacillus. rhamnosus* has significant beneficial effects, such as a positive role in obesity and activities against resistant bacteria (vancomycin‐resistant enterococci). *Lactobacillus. reuteri* naturally colonizes the digestive tract, especially after consumption of dairy products. It is one of the *Lactobacillus* strains that can be transmitted to infants via breast milk.^3^, ^5^ The antimicrobial activity through the production of reuterin, an antibiotic‐like agent, is absolutely remarkable in this strain.^3^ *Lactobacillus brevis* is another *Lactobacillus* strain with remarkable effects on intestinal homeostasis and inhibitory effects on intestinal inflammation.^6^

As mentioned earlier, modulation of the immune system and anti‐inflammatory effects are among the beneficial effects of probiotics. These processes are critical for the management and control of inflammatory diseases, including inflammatory bowel disease (IBD). Both Th‐1 and Th‐2 activities have been observed in Crohn's disease (CD) and ulcerative colitis (UC), the two subtypes of IBD. Therefore, in IBD, there is often an overproduction of inflammatory cytokines leading to inflammation.^7^ IBD is considered a chronic relapsing and remission disease. The use of agents with anti‐inflammatory activity to relieve the symptoms that occur in the relapsing phase could be helpful in controlling IBD. In addition, the use of a long‐lasting therapeutic agent that could influence the duration of the remission phase is also crucial.^8^ Probiotics not only have an anti‐inflammatory effect but often continue to act for up to 3 weeks after the end of treatment and therefore appear to be a suitable means of prolonging the duration of remission and controlling symptoms in patients with IBD.^9^

Several signaling pathways, such as Janus kinase/signal transducer and activator of transcription (JAK/STAT) and nuclear factor kappa‐light‐chain‐enhancer of activated B cells NF‐κB, are involved in the production and function of cytokines and therefore play roles in the inhibition or progression of IBD.^10^ Probiotics, as anti‐inflammatory agents, could have beneficial effects on modulating these signaling pathways and thus improve the inflammatory status in patients with IBD. Although our previous studies have shown phenotypic anti‐inflammatory effects of probiotics,^11^ evaluation of the exact molecular mechanisms of probiotics could elucidate the ameliorative process more precisely. On the other hand, it would be important to find out whether probiotics could have beneficial effects in both phases of IBD, that is, relapse and remission. In other words, if probiotics have positive modulatory effects on both phases, it could be said that these live agents could be consumed as preservatives and also as complementary treatments. Here, we aimed to investigate the efficacy of *Lactobacillus* spp. in modulating JAK/STAT and NF‐κB signaling pathways to understand how probiotics might play an effective role in reducing inflammation before, during, and after the presence of an inflammatory state.

## 2 MATERIALS AND METHODS

### 2.1 Preparation of bacterial strain

In the current study, the in vitro assay was conducted to evaluate the effects of probiotics on the NF‐κB and JAK/STAT signaling pathways. Four *Lactobacillus* spp. including *L*. *plantarum*, *L*. *rhamnosus*, *L*. *brevis*, and *L*. *reuteri* were isolated from the natural gut microbiota of fecal samples from 53 healthy volunteers aged 1−36 years, and the probiotic and phenotypic characteristics of these strains were previously investigated.^11^ Bacteria were inoculated into De Man, Rogosa and Sharpe agar (MRS) broth with 0.05% l‐cysteine and incubated at 37°C for 20 h. After centrifugation at 8000 rpm for 5 min, the pellets were diluted with the antibiotic‐free MRS medium containing 10% fetal bovine serum (FBS), and the optical density (OD) was adjusted to 0.08 (0.5 McFarland). To make the probiotic cocktail, an equal amount of *Lactobacillus* spp. was mixed and the final concentration was adjusted to 0.08 OD using a spectrophotometer at 600 nm. For treatments, the culture pellet was collected and diluted in RPMI‐1640 with 10% FBS without antibiotics to obtain the final OD concentration of 0.08 at 600 nm.

For induction of inflammation sonicated pathogen (SP) was used. For preparation, Enterotoxin‐producing *Escherichia coli* (ETEC) and *Salmonella typhimurium* (ST) were cultured in Luria−Bertani (LB) broth (Thermo Fisher Scientific). After that, heat‐killed cultures (100°C for 10 min) were sonicated (10 rounds, 1 min/round), and cell debris was centrifuged (1700*g*, 15 min, 4°C). All methods were performed according to the relevant guidelines and regulations, and ethical approval for the previous study was obtained from the Pasteur Institute of Iran Committee (IR.PII. REC.1398.060). Signed informed consent was obtained from all participants.

## 3 PROBIOTIC TREATMENT OF HT‐29 CELL LINE

### 3.1 Cell culture procedure

The human colon adenocarcinoma cell line HT‐29 was purchased from the Cell Bank of the Pasteur Institute of Iran. For HT‐29 cell line, RPMI‐1640 (Thermo‐Gibco) supplemented with 10% fetal bovine serum (Biochrom) and 1% penicillin−streptomycin (Sigma‐Aldrich) was used. Cells were then separated with 0.25% trypsin‐EDTA (Gibco), washed twice with PBS, and counted. The cell suspension was centrifuged and the precipitate was diluted with RPMI‐1640 and 2 × 10^5 ^cells per well were seeded.

### 3.2 SP and *Lactobacillus* spp. treatments

HT‐29 cells were exposed to *Lactobacillus* spp. (all four species in a cocktail form) and SP. The components are as follows: sonicated enterotoxigenic *E. coli* (SP‐ETEC), sonicated *Salmonella typhi* (SP‐ST), and *Lactobacillus* spp. To study the effect of probiotics before, during, and after the inflammatory state, *Lactobacillus* spp. were added as follows: (a) first, *Lactobacillus* spp. was added to the HT‐29 cell line and, after 6 h, SP‐ETEC and SP‐ST were added to cause inflammation (LP). This treatment was used to observe the effect of *Lactobacillus* spp. before the inflammatory condition was triggered. (b) First, SP‐ETEC and SP‐ST were added to the HT‐29 cell line and, after 6 h, *Lactobacillus* spp. was added to determine the supposed effects (PL). This treatment was used to see the effect of *Lactobacillus* spp. after triggering the inflammation. (c) *Lactobacillus* spp. and SP (SP‐ETEC and SP‐ST) were simultaneously added to the HT‐29 cell line (P + L). This treatment was used to show the effect of *Lactobacillus* spp. while the inflammatory condition was induced. In the next step, each well was washed twice with PBS to remove the nonadherent bacteria. These treatments were performed in duplicate and the cell culture was maintained at 37°C and 5% CO_2_ for up to 48 h. Determination of MOI was performed as previously indicated.^12^

### 3.3 RT‐PCR of inflammatory signaling pathway genes

An RNA extraction kit (Roche) was used to extract total RNA according to the manufacturer's instructions. The amount and quality of purified RNA were determined using a NanoDrop1000 UV‐Vis spectrophotometer (measuring absorbance at 260/280 nm). The cDNA template was synthesized using the cDNA synthesis kit (Yekta Tajhiz) according to the manufacturer's instructions. The online website Primer Bank (http://pga.mgh.harvard.edu/primerbank) was used to select quantitative real‐time polymerase chain reaction (qPCR) primers (Table 1). All reactions were performed in duplicate. The formula RQ = 2^‐ΔΔ^ *C*_t_ was used to obtain the relative gene expression in the comparative CT method.^13^ The appropriate internal control gene, glyceraldehyde‐3‐phosphate dehydrogenase (*gapdh*), was selected as the housekeeping gene to normalize the data. To evaluate the mRNA quantification of the studied genes, the ABI step one plus detection system (Applied Biosystems Corp.) and SYBR Green Master Mix (Amplicon Bio) were used.

**Table 1:** Primer sequences used in this study.

Gene	Primer sequence (5′ > 3′)	Primer bank ID	Product size (bp)
STAT1 F	CGGCTGAATTTCGGCACCT	189458859c3	81
STAT1 R	CAGTAACGATGAGAGGACCCT		
STAT2 F	CTGCTAGGCCGATTAACTACCC	291219923c3	87
STAT2 R	TCTGATGCAGGCTTTTTGCTG		
STAT3 F	ACCAGCAGTATAGCCGCTTC	47080104c2	124
STAT3 R	GCCACAATCCGGGCAATCT		
STAT4 F	GCTTAACAGCCTCGATTTCAAGA	345110659c2	91
STAT4 R	GAGCATGGTGTTCATTAACAGGT		
STAT5 F	CGACGGGACCTTCTTGTTG	221316717c3	80
STAT5 R	GTTCCGGGGAGTCAAACTTCC		
STAT6 F	CGAGTAGGGGAGATCCACCTT	296010867c2	92
STAT6 R	GCAGGAGTTTCTATCAAGCTGTG		
JAK1 F	CTTTGCCCTGTATGACGAGAAC	102469033c1	101
JAK1 R	ACCTCATCCGGTAGTGGAGC		
JAK2 F	ATCCACCCAACCATGTCTTCC	223671934c2	121
JAK2 R	ATTCCATGCCGATAGGCTCTG		
JAK3 F	CTGCACGTAGATGGGGTGG	189095272c2	78
JAK3 R	CACGATCAGGTTGGACTTTTCT		
TYK2 F	GAGATGCAAGCCTGATGCTAT	187608614c1	76
TYK2 R	GGTTCCCGAGGATTCATGCC		
RIP2 F	GCCCTTGGTGTAAATTACCTGC	93141034c2	138
RIP2 R	GGACATCATGCGCCACTTT		
NEMO F	AAGAGCCAACTGTGTGAGATG	142381344c1	69
NEMO R	TTCGCCCAGTACGTCCTGA		
TIRAP F	GACCCCTGGTGCAAGTACC	89111123c2	133
TIRAP R	CGACGTAGTACATGAATCGGAG		
IRAK4 F	CTTGGATGGTACTCCACCACT	223671887c3	76
IRAK4 R	AAAATTGATGCCATTAGCTGCAC		

Abbreviations: IRAK, Interleukin 1 Receptor Associated Kinase; JAK, Janus kinase; NEMO: Nuclear factor‐kappa B Essential Modulator; RIP, receptor interacting protein; STAT, signal transducer and activator of transcription; TIR‑associated Protein; TYK, tyrosine kinase.

### 3.4 Cytokine assays

To determine the phenotypic effects of probiotic treatment on inflammation reduction, cytokine production was assessed by ELISA assay. After SP and *Lactobacillus* spp. treatments, the supernatant of the cell culture was centrifuged at 6000 rpm, and the supernatant was collected to evaluate the production of proinflammatory cytokines, including interleukin (IL)‐6 and IL‐1β.

### 3.5 Statistical analysis

Graphs and statistical analysis of the data were performed using SPSS (ver. 25) and GraphPad Prism software to compare variables of different groups. Statistical differences between multiple groups were determined using ordinary one‐way analysis of variance (ANOVA). *p* < .05 were considered statistically significant. The results were presented as mean ± SD.

## 4 RESULTS

In the present study, we investigated the anti‐inflammatory effect of live *Lactobacillus* spp. The cocktail form with four different species was used to enhance the anti‐inflammatory effect of the probiotic strains. In addition, the SP was used to induce the inflammatory state because bacterial components, including lipopolysaccharide (LPS) and flagellin of gram‐negative bacteria, are released during the sonication process and these components could trigger inflammation via triggering inflammatory signaling pathways. *Lactobacillus* spp.‐treated HT‐29 cells were compared with C24 and C48 (negative controls) and SP24 and SP48 (positive controls) to evaluate the efficacy of *Lactobacillus* spp. in up or downregulating the studied genes. It should be noted that the concept of treatment stages (phases) refers to taking probiotics before, after, and during the induction of inflammation.

### 4.1 The results of *STAT* genes expression

**Figure 1:**
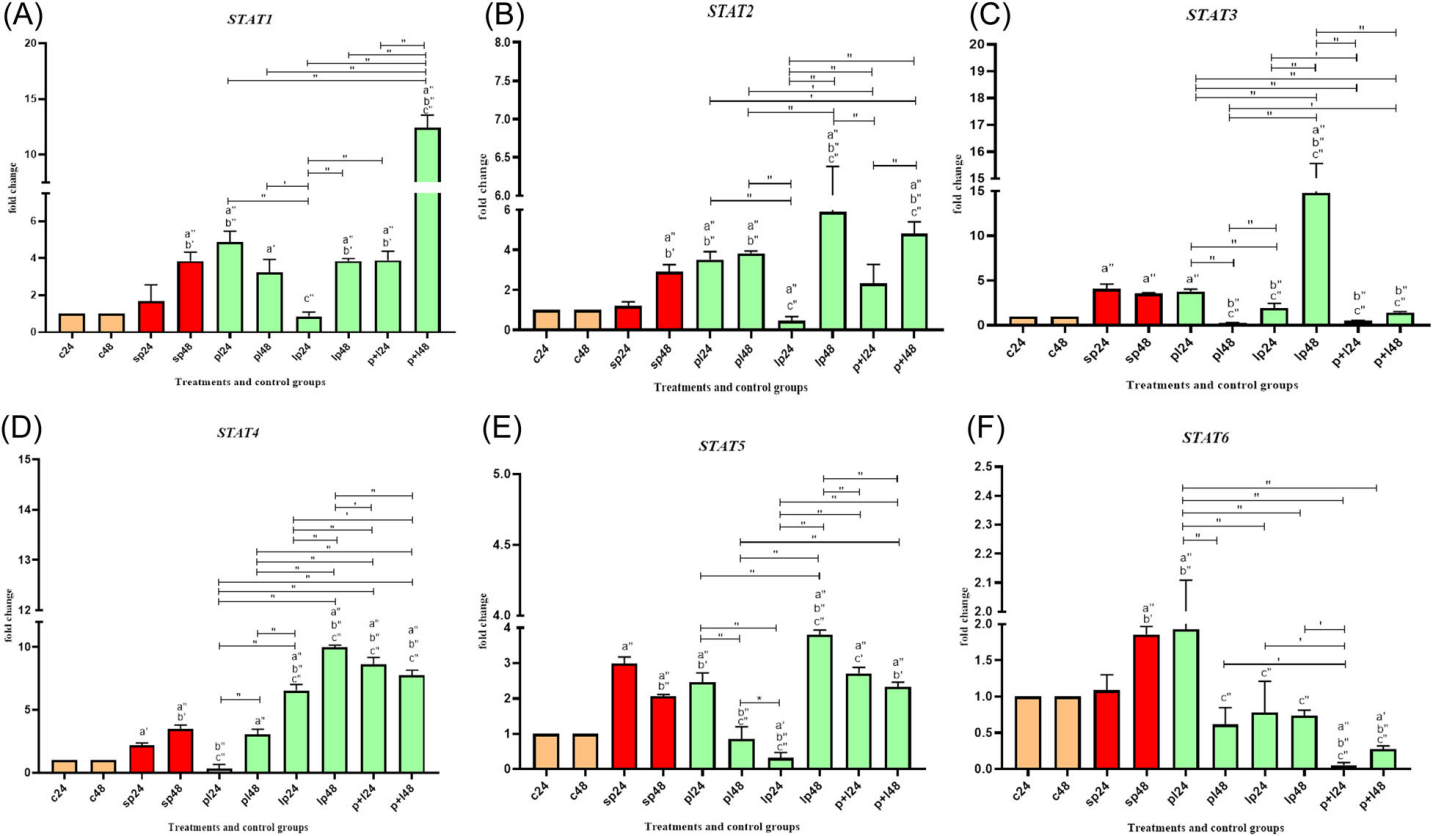
Relative gene expression (mean fold change) of *STAT* genes in the different groups of treatments (A‐F for STAT 1‐6). Data were represented as mean ± SD. Data were considered as statistically significant when *p* < .05 ('*p* < .05, "*p* < .001). Letter a indicates the relatedness between C24 and C48 with other treatments, letter b shows the relatedness between sp24 and other treatments, and letter c shows the relatedness between sp48 with other treatments. The relatedness between other treatments is shown with bracket.

Data on *STAT* gene expression are shown in Figure 1. Compared to negative controls, the expression level was increased by SP (*p* < .05). However, both up and downregulation was observed for *STAT* genes after probiotic treatments compared to positive controls in a different state of inflammation.

Comparative analysis of *STAT1*, *STAT2*, and *STAT4* gene expressions showed that *Lactobacillus* spp. treatment upregulated gene expression at most treatment stages. However, LP24 and PL24 downregulated the expression level in *STAT1, STAT2*, and *STAT4*, respectively (*p* < .001). In *STAT5*, again LP24 could significantly decrease the expression level (*p* < .05), while LP48 was able to significantly increase the expression level (*p* < .0001).

For *STAT3* and *STAT6*, the general trend was downward in most treatment phases. In particular, P + L24 in both genes and PL48 in *STAT3* had the greatest effect on reducing expression levels (*p* < .05).

### 4.2 The results of *JAK* genes expression

**Figure 2:**
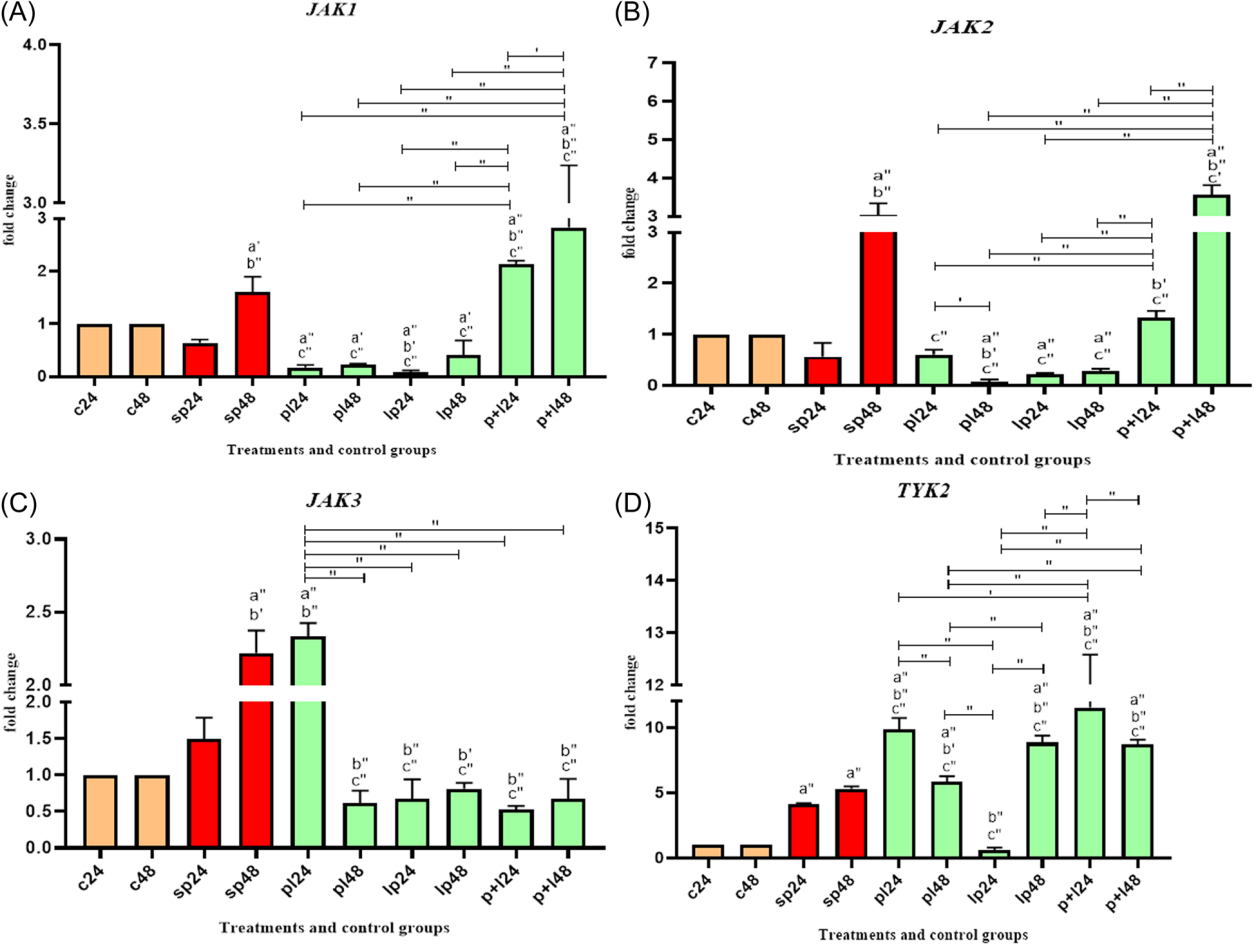
Relative gene expression (mean fold change) of *JAK* genes in the different groups of treatments (A‐D for JAK 1, JAK 2, JAK 3 and TYK 2 genes). Data were represented as mean ± SD. Data were considered as statistically significant when *p* < .05 ('*p* < .05, "*p* < .001). Letter a indicates the relatedness between C24 and C48 with other treatments, letter b shows the relatedness between sp24 and other treatments, and letter c shows the relatedness between sp48 with other treatments. The relatedness between other treatments is shown with bracket. TYK, tyrosine kinase

Data on *JAK* expressions are shown in Figure 2. Comparative analysis of *JAK* gene expression between SP and negative controls showed that SP‐ETEC and SP‐ST could significantly increase gene expression.

Comparative analysis of *JAK1* showed that *Lactobacillus* spp. could downregulate the expression level both before and after causing inflammation (*p* < .0001). However, when *Lactobacillus* spp. were added simultaneously with the SP, the expression level was significantly increased (*p* < .0001).

For *JAK2*, *Lactobacillus* spp. was able to downregulate the expression level before and after triggering inflammation compared to SP48 (*p* < .0001). When *Lactobacillus* spp. was added simultaneously with the sonicated pathogen, the result was not homogeneous. *Lactobacillus* spp. downregulated the expression level in the first 24 h of treatment, but gene expression was increased after 48 h. In *JAK3*, the general trend was downward in all treatment phases, except for PL24.

Comparative analysis of tyrosine kinase (*TYK)2* gene expression showed that *Lactobacillus* spp. treatment upregulated gene expression at most treatment stages. However, when *Lactobacillus* spp. was added to the HT‐29 cell line before inflammation induction, the expression level was decreased after 24 h of treatment (LP24) (*p* < .0001). Adding *Lactobacillus* spp. together with SP (during inflammation state) had the most effects on increasing the expression level of the *TYK2* gene (P + L24) (*p* < .05).

### 4.3 The results of inflammatory genes expression

**Figure 3:**
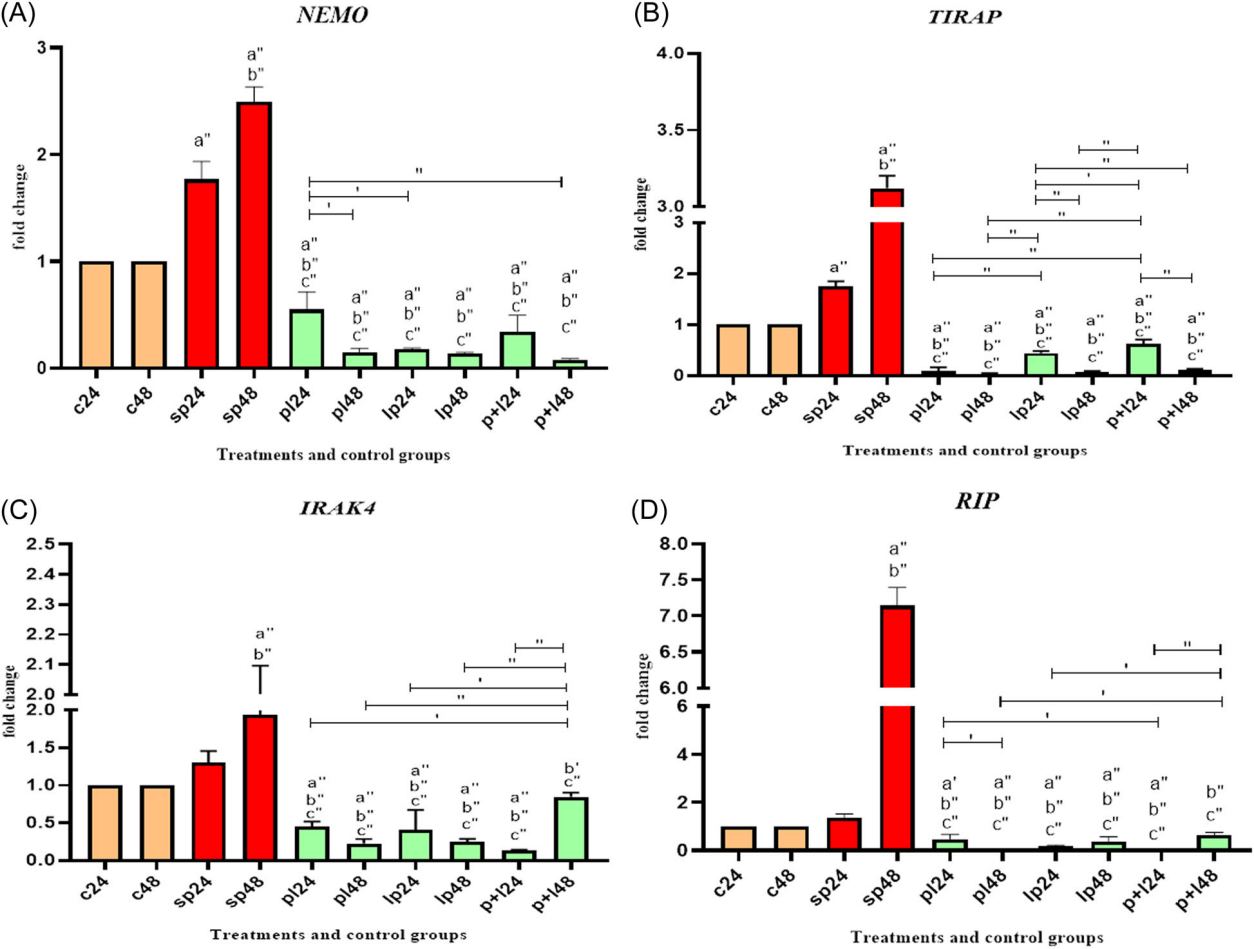
Relative gene expression (mean fold change) of inflammatory genes in the different groups of treatments (A‐ D for NEMO, TIRAP, IRAK 4 and RIP genes). Data were represented as mean ± SD. Data were considered as statistically significant when *p* < .05 ('*p* < .05, "*p* < .001). Letter a indicates the relatedness between C24 and C48 with other treatments, letter b shows the relatedness between sp24 and other treatments, and letter c shows the relatedness between sp48 with other treatments. The relatedness between other treatments is shown with bracket. IRAK, Interleukin 1 Receptor Associated Kinase; NEMO, Nuclear factor‐kappa B Essential Modulator; TIRAP, TIR‑associated Protein.

Inflammatory gene expression data are shown in Figure 3. Comparative analysis of inflammatory gene expression, including Nuclear factor‐kappa B Essential Modulator (*NEMO)*, TIR‑associated Protein (*TIRAP)*, Interleukin 1 Receptor Associated Kinase (*IRAK)*, and receptor interacting protein (*RIP)* between SP and negative controls showed that SP‐ETEC and SP‐ST could significantly increase gene expression, especially after 48 h. *Lactobacillus* spp. treatment had significant reducing effects at all treatment stages. In other words, it could reduce expression before, after, and during inflammation induction. It should be noted that *Lactobacillus* spp. treatments reduced the expression of *TIRAP* and *RIP* to zero.

### 4.4 The result of proinflammatory cytokines production

**Figure 4:**
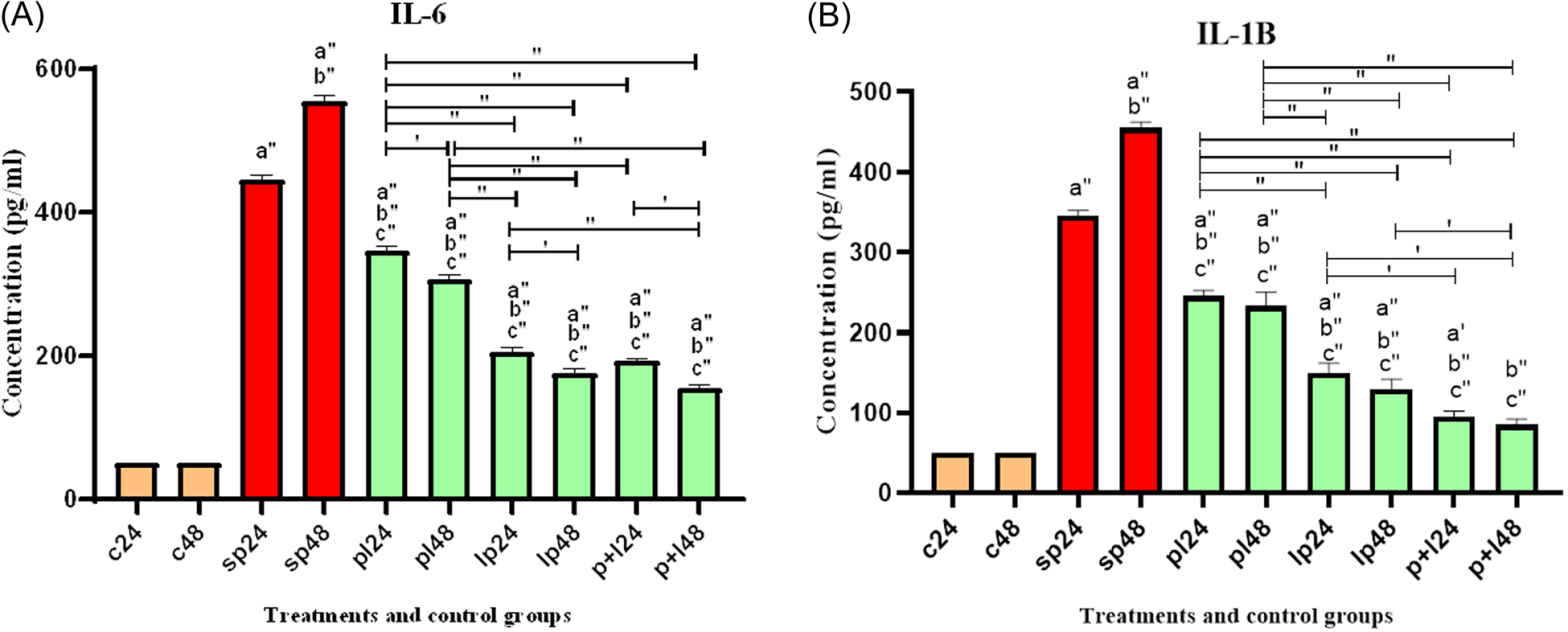
Different levels of concentrations of proinflammatory cytokines (A and B for IL‐6 and IL‐1 B). Data were represented as mean ± SD. Data were considered as statistically significant when *p* < .05 ('*p* < .05, "*p* < .001). Letter a indicates the relatedness between C24 and C48 with other treatments, letter b shows the relatedness between sp24 and other treatments, and letter c shows the relatedness between sp48 with other treatments. The relatedness between other treatments is shown with bracket.

The result of proinflammatory cytokines production are shown in Figure 4. Cytokine production was significantly higher after SP treatments. However, treatment with *Lactobacillus* spp. significantly decreased cytokine production before, after, and during inflammation. It appears that the addition of *Lactobacillus* spp. along with the SP (during inflammation) had the greatest effect on reducing inflammatory cytokines.

## 5 DISCUSSION

IBD as a chronic recurrent disease affects various aspects of the patient. The exact cause or pathogenesis of IBD is not yet fully understood, but dysbiosis of the gut microbiome along with dysregulation of the immune system could lead to inflammation, which is usually observed in patients with IBD. One of the biggest challenges in IBD patients is the fact that the probability of relapse within the next year is 70%−80% in patients with active disease. Thus, if therapeutic choices could prolong the period of remission in the patient, this inactive phase could persist for the next few years. Patients with CD usually suffer more severe symptoms than patients with UC, including hypovolemia, protein‐calorie malnutrition, and anemia. However, it takes 2 years after the initial diagnosis for the first relapse and surgical therapies are usually not used until 10 years after the initial diagnosis^14^ thus, these time periods appear to be long enough that anti‐inflammatory agents could reduce inflammation and improve symptoms. The severity of IBD may progress to affect other organs such as joints, skin, eyes, and to a lesser extent lung, liver, and pancreas.^15^ Therefore, patients with IBD are strongly recommended to use therapeutic agents, especially those with the least side effects, including probiotics, to reduce the risk of surgical intervention, limit the symptoms of IBD, and prolong the time of remission.^16^ Signaling pathways as the messenger system in cell function could show the effects of environmental stimulators in the improvement of cell hemostasis and as mentioned earlier, we have seen the phenotypic anti‐inflammatory effects of our probiotic strains in an in vivo study.^11^ Therefore, we wanted to find out the effects of probiotics on gene expression of signaling pathways that could affect the inflammatory response in the gut including NF‐κB and JAK/STAT pathways.

The results of the cytokine assay (reduction of anti‐inflammatory cytokines) in the present study are consistent with the results of our previous study that showed the anti‐inflammatory effect of *Lactobacillus* in an in vivo model.^11^ In the present study, *Lactobacillus* spp. treatments in all versions of inflammation induction had significant reduction effects, especially when probiotics and sonicated pathogens were added simultaneously. These phenotypic results were confirmed by the molecular investigation. The results of NF‐κB expression levels were homogeneous. The addition of  sonicated pathogens could significantly increase the expression level. This evidence could show the probable role of pathogenic components to cause inflammation. However, treatment with *Lactobacillus* spp. had a significant reducing effect. *Lactobacillus* spp. had a reducing effect on the expression of inflammatory genes before, after, and during the initiation of inflammation. NF‐κB is considered one of the main mediators of inflammatory responses and can induce the expression of several proinflammatory genes.^17^ Reducing the expression of these genes could therefore have a beneficial effect on reducing inflammation.

The results of the JAK/STAT pathway in the current study were different. In other words, *Lactobacillus* spp. had both decreasing and increasing effects on expression levels. However, these results are also consistent with the phenotypic results and the anti‐inflammatory properties of the probiotics. In the current study, the overall trend of expression of some STATs, including *STAT3* and *STAT6*, was downward. In general, downregulation of this pathway has remarkable effects on reducing inflammation. Several studies have reported that reducing the expression of STAT3, for example, can lead to a reduction in IL‐6 and thus contribute to the reduction of the inflammatory state.^18^, ^19^ STAT6 is another STAT with increased phosphorylation in patients with UC. Studies have reported that colitis is limited in STAT 6^−/−^ mice and that deficiency of STAT6 may be helpful in improving IBD.^20^ On the contrary, the expression of other *STATs*, including *STAT1*, *STAT2*, *STAT4*, and *STAT5*, was approximately increased. These data could be confirmed by other reports. The overall function of JAK/STAT in controlling IBD is complicated. For example, Rauch et al.^21^ reported that a microarray study of biopsies revealed that *STAT1* expression might be elevated in CD, but this condition could not usually be observed in patients with UC. The association between STATs, including STAT1, and anti‐inflammatory cytokines, including the IL‐10 superfamily, may be one of the reasons supporting the beneficial effects of upregulating *STAT1* in the treatment of inflammatory diseases such as IBD.^22^ The same condition could be observed for *STAT5*. IL‐22 as one of the anti‐inflammatory cytokines could activate STAT1, STAT5, JAK1, and TYK2 and mediate its function.^23^ Several studies also addressed the different roles of other STATs, including STAT2 and STAT4. Yang et al.^24^ reported that STAT4 may have beneficial effects on the control of IBD by inhibiting Th17 accumulation and promoting repair of the damaged intestinal epithelium by inhibiting activation of the IL17α/IL17 promoter. STAT2 also plays different roles in the immune system. Because it can be activated by type I interferons (IFNs), it plays an important role in immunomodulation, which is usually impaired in patients with IBD.^25^ All of these data may support the beneficial effects of upregulation of *STATs* genes.

The results of JAKs also showed remarkable data on the control of inflammation. The overall trend for *JAK1*, *JAK2*, and *JAK3* was downward. Targeting JAKs to control IBD has been discussed in several studies. In other words, any agent that has an inhibitory effect on the activities of JAK could control the inflammatory state in patients with IBD.^26^ However, the results of *TYK2* seem to be the opposite. The general trend in the expression of TYK2 was increasing. TYK2, together with the STATs component, might be associated with several cytokines. IL‐10 and IFN‐λ are two of the cytokines with anti‐inflammatory properties associated with TYK2.^27^ Therefore, upregulation might be helpful for the activity of anti‐inflammatory status.

In conclusion, evaluating the precise molecular effects of *Lactobacillus* spp. on signaling pathways could provide clear insight into how probiotics modulate and reduce inflammation. One of the most important issues is understanding the anti‐inflammatory properties of probiotics and clarifying whether probiotics could be used as a preservative or as a therapeutic. Finding the easiest way to both prevent and relieve the symptoms of IBD could be of great importance to IBD patients. Since we use *Lactobacillus* spp. as pre, post, and cotreatment, and observation of its beneficial effects on all three variants, the results may suggest that these probiotic strains, in turn, could prevent and treat the severity of IBD.

## AUTHOR CONTRIBUTIONS

*Performed the experiments*: Shadi Aghamohammad, Seyedeh Tina Miri, and Saeideh Najafi. *Data analysis*: Amin Sepehr, Mahdi Rohani, and Shadi Aghamohammad. *Writing of the manuscript*: Shadi Aghamohammad and Amin Sepehr. *Revised manuscript*: Mohammad R. Pourshafie and Mahdi Rohani. *Conceived and designed the experiments*: Mohammad R. Pourshafie and Mahdi Rohani.

## CONFLICT OF INTEREST

The authors declare no conflict of interest.

## ETHICS STATEMENT

The experimental protocols were established following the Declaration of Helsinki and approved by the ethics committee of Pasteur Institute of Iran (IR.PII.REC.1398.060). Signed informed consent was obtained from all participants.

## Data Availability

The data sets generated during and/or analyzed during the current study are available from the corresponding author on reasonable request.
